# Monolayer nanosheets formed by liquid exfoliation of charge-assisted hydrogen-bonded frameworks†

**DOI:** 10.1039/d0sc06906j

**Published:** 2021-01-14

**Authors:** Joshua Nicks, Stephanie A. Boer, Nicholas G. White, Jonathan A. Foster

**Affiliations:** Department of Chemistry, University of Sheffield Sheffield UK jona.foster@sheffield.ac.uk; Research School of Chemistry, The Australian National University Canberra ACT 2600 Australia nicholas.white@anu.edu.au

## Abstract

Hydrogen-bonded organic frameworks (HOFs) are a diverse and tunable class of materials, but their potential as free-standing two-dimensional nanomaterials has yet to be explored. Here we report the self-assembly of two layered hydrogen-bonded frameworks based on strong, charge-assisted hydrogen-bonding between carboxylate and amidinium groups. Ultrasound-assisted liquid exfoliation of both materials readily produces monolayer hydrogen-bonded organic nanosheets (HONs) with micron-sized lateral dimensions. The HONs show remarkable stability and maintain their extended crystallinity and monolayer structures even after being suspended in water at 80 °C for three days. These systems also exhibit efficient fluorescence quenching of an organic dye in organic solvents, superior to the quenching ability of the bulk frameworks. We anticipate that this approach will provide a route towards a diverse new family of molecular two-dimensional materials.

## Abstract 2

We report the liquid-phase ultrasonic exfoliation of two layered hydrogen-bonded frameworks into monolayer, micron-sized, and water-stable nanosheets (HONs) connected purely by hydrogen-bonding interactions.

## Graphical abstract

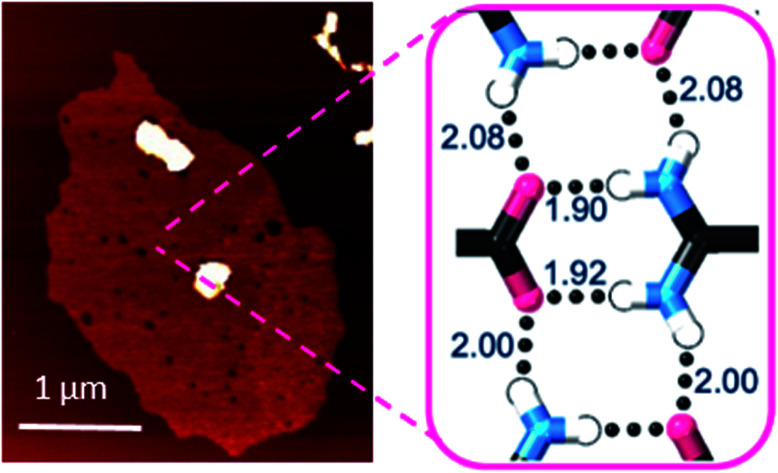

## Introduction

Liquid exfoliation has been used to convert a diverse range of layered materials into two-dimensional nanosheets. This simple and scalable approach requires materials with strong in-layer interactions but weak inter-layer interactions to create free-standing single and few-layer nanosheets with high aspect ratios. Early studies focussed on two-dimensional materials formed using either covalent bonds, such as in graphene and boron nitride,^1,2^ or ionic bonding such as in molybdenum disulphide and layered double hydroxides.^3,4^ However, more recent examples have shown this approach can be adapted to exfoliate supramolecular structures formed using dynamic covalent bonds, to form covalent organic framework nanosheets (CONs),^5,6^ or coordination compounds, to form metal–organic framework nanosheets (MONs).^7,8^ The dynamic chemistry of these materials allows them to be produced under milder conditions than inorganic two-dimensional materials and their molecular nature allows their structure and properties to be more easily modified. The high surface area, aspect ratios, and nanoscopic dimensions of these nanosheets combined with their diverse and tunable chemistry make them ideal for a wide range of sensing,^9,10^ catalysis,^11–13^ electronics,^14,15^ and separation applications.^16–18^ However, despite intensive research into all these materials, the formation of monolayer nanosheets with high aspect ratios in good yields remains a challenge.

Hydrogen-bonds can play a varied range of roles within two-dimensional materials. Most commonly, they are located between layers providing relatively weak interactions that interact strongly with polar solvents aiding their exfoliation and dispersion. For example, interlayer hydrogen-bonding has been used to improve the properties of 2D COFs.^19–21^ More relevantly, a variety of nanosheets containing hydrogen-bonds within the layers have also been reported. For example, liquid–air interfaces have been used to direct the self-assembly of a number of carboxylic acid and amide functionalised molecules to create ultrathin nanosheets with high aspect ratios.^22–24^ Other reports have shown that naphthalene-diimide derivatives can self-assemble in solution to form nanosheets through face to face stacking of the monomeric units.^25,26^ A range of urea-based gelators have also been shown to self-assemble into 2D nanosheets rather than 1D tapes.^27,28^ Cucurbituril-based nanosheets down to monolayer thickness have been reported which are held together through a combination of π–π stacking, CH/π interactions and CH/C=O hydrogen-bonding.^29^ A bis-acylurea based organogelator was found to stack through hydrogen-bonds in two-dimensions to form nanosheets, rather than the usual 1-dimensional structure. A range of self-assembled peptides, and block-co-polymer-based nanosheets have also been reported.^30–33^ All of these nanosheets are based on relatively weak hydrogen-bonding interactions in combination with hydrophobic aromatic or aliphatic groups which in most cases drive nanosheet formation through the hydrophobic effect.^34,35^ These nanosheets have also all been formed “bottom-up” through dynamic self-assembly processes in solution. An array of 2D hydrogen-bonded materials on surfaces have also been demonstrated using a variety of building motifs, though these are not free-standing.^36,37^ We are only aware of a few examples of “top-down” formation of nanosheets containing hydrogen-bonds within the layers, but in these examples hydrogen-bonds are only formed in one dimension and co-ordination bonds,^38^ covalent bonds,^39^ or pi-stacking,^40^ hold the nanosheets together in the other. To our knowledge there are no examples of nanosheets connected in two-dimensions exclusively through strong hydrogen-bonding interactions.

Hydrogen-bonded organic frameworks (HOFs) are a class of porous supramolecular materials assembled through intermolecular hydrogen-bonding interactions.^41,42^ Like MOFs and COFs, in order to create porous structures these materials typically rely on rigid aromatic linkers and strong directional hydrogen-bonding groups. HOFs possess a distinct set of properties in comparison to most other framework materials, including ease of synthesis, solution processability, and self-healing.^43^ While many HOFs are constructed through hydrogen-bonding between neutral self-complementary groups,^41,42^ some systems use charge-assisted hydrogen-bonds,^44–47^ which may lead to more robust materials. In particular, amidinium cations and carboxylate anions are known to form strong charge-assisted hydrogen-bond donor–acceptor pairs, and we have shown that 3D frameworks prepared from this pair show high stability, including to prolonged boiling in water, and heating in polar solvents such as DMSO.^48,49^

Although a wide variety of layered HOFs have previously been reported,^50–52^ to our knowledge there are currently no studies investigating their exfoliation to form free-standing hydrogen-bonded framework nanosheets (HONs). This is perhaps because it is not necessarily obvious that materials formed using relatively weak intra-layer hydrogen-bonding interactions would survive the high shear forces associated with liquid exfoliation. In other examples of nanosheets that include hydrogen-bonds within the layers, the hydrogen-bonds are typically shielded from polar solvent by hydrophobic groups. However, the rigid structures of HONs mean that exfoliation to form monolayer nanosheets would directly expose the hydrogen-bonds, making them accessible to solvent molecules and potentially leading to dissolution. HONs therefore currently represent a new and unexplored class of two-dimensional materials.

In this work, we report the formation of two layered hydrogen-bonded frameworks connected in two-dimensions through strong charge-assisted hydrogen-bonds between amidinium and carboxylate groups. We hypothesized that the strong in-layer interactions within these frameworks would enable them to undergo ultrasonic exfoliation to produce nanosheets. Remarkably, both materials were found to readily exfoliate to form micron-sized single-layer HONs in good yields. Furthermore, the nanosheets showed impressive stability in water and the ability to quench fluorescence of organic dyes emphasising the potential of HONs as a distinct new class of 2D materials.

## Results and discussion

### Synthesis of layered hydrogen-bonded frameworks

**Fig. 1:**
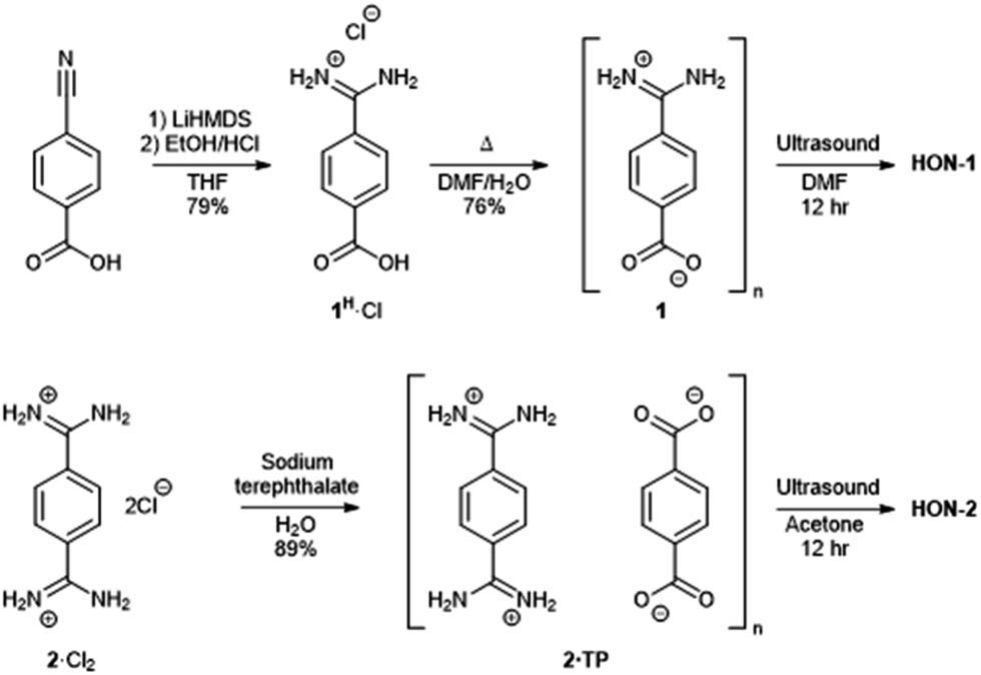
Scheme showing the synthesis of hydrogen-bonded frameworks **1** and **2·TP** and their exfoliation to form **HON-1** and **HON-2**.

Two hydrogen-bonded systems incorporating amidinium and carboxylate synthons either within a single mixed linker (**1**) or as a co-crystal (**2·TP**) were prepared using simple high yielding syntheses, shown in Fig. 1. Commercially available 4-cyanobenzoic acid was converted to the amidinium compound **1**^H^·Cl using excess lithium bis(trimethylsilyl)amide (LiHMDS) followed by acidic work-up. Crystals of the zwitterionic form of 4-amidiniumbenzoate (**1**) were obtained by heating in DMF/H_2_O at 120 °C for two days. Co-crystals of **2·TP** were obtained on a 300 mg scale by carefully layering an aqueous solution of bis(amidinium) **2**·Cl_2_ with an aqueous solution of sodium terephthalate (Na_2_·**TP**).^53^

**Fig. 2:**
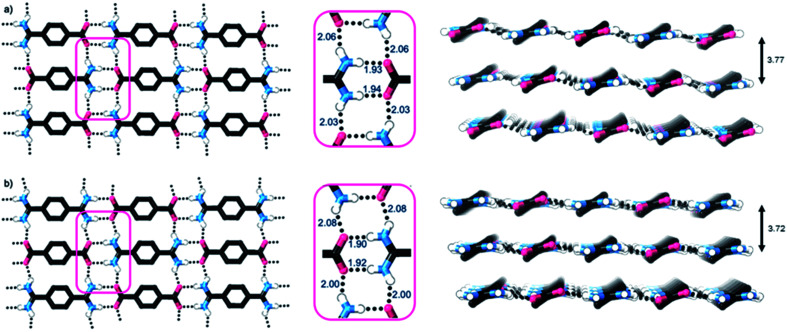
(a) and (b) Views of the X-ray crystal structures of **1** and **2·TP** respectively, showing the 2D hydrogen bonded sheets (C–H hydrogen atoms omitted for clarity). Portions of the structures are expanded in pink boxes, which highlight the H-bonding distances (in Å). Views of the stacking of the 2D hydrogen bonded layers in each structure, with interlayer distances, are shown adjacent (C–H hydrogen atoms omitted for clarity).

Single crystal X-ray diffraction studies show that the crystal structures of both **1** and **2·TP** are layered structures with strong in-layer hydrogen-bonding and weak inter-layer interactions. As shown in Fig. 2, both HONs consist of 1D hydrogen bonded chains, which propagate through short N–H⋯O hydrogen-bonds along the axis of the 1,4-disubstituted phenyl groups [H⋯O = 1.93, 1.94 Å in **1** and 1.88, 1.89 Å in **2·TP**]. The 1D chains are linked together through additional N–H⋯O hydrogen bonds perpendicular to this, which are slightly longer [H⋯O = 2.03, 2.06 Å in **1**, and 2.01, 2.07 Å in **2·TP**]. Overall, this assembles both structures into 2D hydrogen-bonded sheets.

**Fig. 3:**
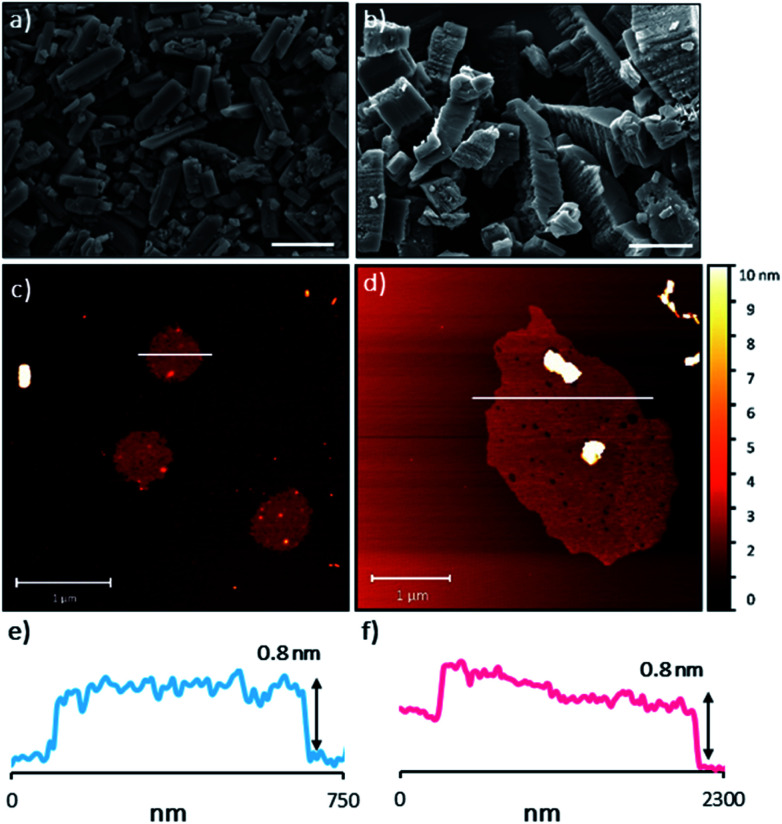
(a) and (b) SEM images of bulk **1** and **2·TP** respectively. Scale bars represent 10 μm. (c) and (d) selected AFM topographical images of the nanosheets formed from exfoliation of **1** and **2·TP** respectively. (e) and (f) height profiles of the nanosheets shown in (c) and (d) (represented by white bars).

The sheets stack with an interlayer distance of 3.77 and 3.72 Å respectively and there are no contacts between sheets that are shorter than the sum of the van der Waals radii (Fig. 2). The shortest centroid⋯centroid contacts between aromatic rings are >4.9 Å in both **1** and **2·TP**. Furthermore, the donor–acceptor pairs of the amidinium and carboxylate groups stack above/below the phenyl rings of the adjacent layers, indicating an absence of substantial interlayer electrostatic interactions between the charged groups. This lack of any significant inter-layer hydrogen-bonding or electrostatic interactions combined with the non-interdigitated stacking mode of these hydrogen-bonded frameworks makes them ideal candidates for ultrasound-assisted liquid exfoliation. SEM imaging of the crystalline powders (Fig. 3a, b, S11 and S12†) indicates the formation of rectangular prisms approximately 3–20 μm wide, with evidence of layers stacking along the long direction of the crystallites.

### Preparation of HONs by liquid exfoliation

Exfoliation experiments were performed using a previously reported bath sonicator setup which we have used to exfoliate layered MOFs to form MONs.^54,55^ Here, “softer” high frequency (80 kHz) ultrasound is used to minimise fragmentation and samples are stirred in a temperature-controlled bath to minimise hot-spots and ensure reproducibility.^54^ During ultrasonication, shear forces and cavitation exert force on the bulk material and induce exfoliation, after which the solvent–nanosheet interaction must overcome inter-sheet attraction to prevent reaggregation. As such, a range of polar solvents (acetone, acetonitrile, DMF, DMSO, and water) were initially investigated for the exfoliation of both **1** and **2·TP**, as it was hypothesized that these would best penetrate between the layers of each system, leading to enhanced exfoliation and colloidal stabilization.

Samples (5 mg) of **1** and **2·TP** were each suspended in 6 mL of solvent in a sealed vial, and subject to ultrasonication at 80 kHz for 12 hours at temperatures below 18 °C. The samples were then centrifuged at 1500 rpm for 1 hour to remove unexfoliated material and isolate nanosheets in suspension (illustrated in Fig. S13†). Tyndall scattering observations provided qualitative evidence indicating that DMF and acetone were optimal solvents for the exfoliation of **1** and **2·TP** into **HON-1** and **HON-2** respectively, with each exhibiting strong scattering effects (Fig. S14†). The other solvents showed weaker Tyndall scattering, indicating nanoparticle formation but with significantly reduced yields and thus were not further investigated.

Sample suspensions were drop-cast onto hot mica plates for topographical AFM imaging (Fig. 3c and d, S15 and S16†). Size distribution analysis of the recorded images indicated consistently ultrathin thicknesses of approximately 0.8 nm for both systems. The expected thickness of a monolayer is calculated as approximately 0.35 nm from the single crystal data. Graphene has a nominal thickness of 0.34 nm but AFM imaging typically shows thickness of 0.4–1.7 nm which is typically attributed to tip surface interactions, image feedback settings and surface chemistry.^56^ Whilst it is possible that bi- or tri-layer nanosheets (2–3 layers) have been consistently formed, the uniform thickness and lack of step edges is better explained by monolayer formation.

Analysis of the lateral dimensions of the imaged nanosheets indicated lengths between 0.3–1.5 μm for **HON-1**, and 0.9–3.4 μm for **HON-2**. As such, the as prepared HONs possess remarkably large aspect ratios and exposed surface areas as a result. The nanosheets have a consistently flat morphology, with no wrinkling or rolling observed *via* AFM. The smaller HONs are observed to be rounded in shape, whereas the larger >800 nm wide sheets have more irregular shapes. Dynamic light scattering measurements of the nanosheets in colloidal suspension corroborate these size distribution observations (Fig. S17†).

The as-prepared HON suspensions were colloidally stable for approximately two days before precipitation was observed. The nanosheets were removed from suspension by centrifugation at 10 000 rpm for 1 hour. Yields of 10 and 12% were obtained for **HON-1** and **HON-2** respectively. Ultrasonic liquid exfoliation of other supramolecular nanosheets such as MONs and CONs typically produce nanosheets with a range of thicknesses and sub-micron lateral dimensions. Only a few reports of monolayer nanosheets with micron-sized lateral dimensions have been reported,^57,58^ and these are typically formed in much lower overall yields. The improved yields and aspect ratios of HONs relative to other MON and CON systems emphasizes the novelty of these new materials and may be related to the more reversible bonds that assemble them.

**Fig. 4:**
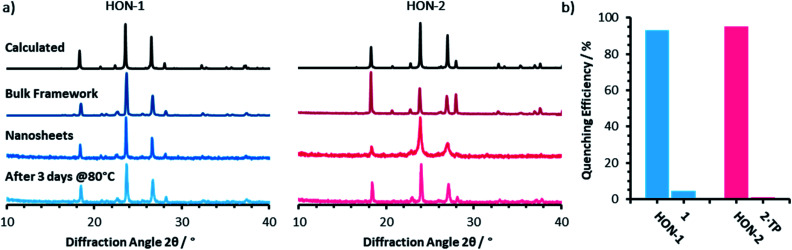
(a) Powder X-ray diffractometry patterns for both the one (blue) and two (pink) component systems, of: the calculated and as synthesised frameworks, nanosheets, and water treated nanosheets. (b) The quenching efficiencies of hydrogen-bonded framework nanosheets and bulk frameworks towards Rhodamine B fluorescence (*λ*_ex_ = 556, *λ*_em_ = 577 nm). Emission titration spectra can be seen in Fig. S21–S24.†

Powder X-ray diffractometry of the bulk crystalline powder, unexfoliated material, and isolated nanosheets indicates that the extended supramolecular structure is maintained post-exfoliation (Fig. 4a). In particular, for the **HON-2** system there is a noticeable reduction in the intensity of peaks corresponding to through-layer planes for the as synthesised HOF (28°, [1, 2, 2]), compared to the in-layer planes (24°, [1, −1, −1]). Line broadening of this type is caused by the finite number of unit cells as crystal dimensions become severely reduced, as described by the Scherrer equation. AFM shows that both systems form monolayer nanosheets so we would expect not to see the out of plane reflections at all. However, as seen with related MON systems,^11,59^ some degree of restacking occurs when samples are dried for analysis. The differences in line broadening therefore most likely reflect different degrees of restacking during sample preparation. It is interesting to note that despite the strong hydrogen-bond donor capabilities of both acetone and DMF, the extended crystallinity is still maintained throughout treatment.

### Water stability and fluorescence quenching activity

Encouraged by the formation of HONs in highly competitive solvents such as DMF, we decided to test the stability of HONs in water. Samples of each HON were heated for 3 days at 80 °C then re-investigated. Powder X-ray diffractometry of both hydrogen-bonded nanosheet systems indicates that the HONs maintain their extended crystallinity (Fig. 4a). This is further supported by the presence of Tyndall scattering in the aqueous suspensions post-treatment (Fig. S18†). AFM imaging of the aqueous suspensions drop-cast onto hot mica shows that the nanosheets maintain their monolayer nature (Fig. S19 and 20†). It is remarkable that atomically thin nanosheets with a supramolecular structure connected only through hydrogen-bonds and without any shielding hydrophobic groups should prove so stable under these conditions. This is particularly notable given that all starting materials dissolve readily in water. This highlights the potential of these materials and of HOFs more generally as stable materials with potential for real-world applications.

To demonstrate the potential for HONs in sensing applications, we investigated the quenching of a fluorescent dye in suspension and compared it to that of the bulk layered framework. Addition of **HON-1** or **HON-2** to a dilute solution of Rhodamine B in acetone resulted in 93 and 95% quenching of the emission respectively (Fig. 4b, S21–S24†). Furthermore, addition of the same mass of respective bulk materials resulted in significantly less effective quenching of the emission, which is attributed to the much higher accessible surface area of the HONs. The nanosheets exhibit no absorption in the UV-visible region beyond 330 nm (acetone's cut-off). This indicates that quenching by the HONs, rather than competitive absorbance of the light, is responsible for the reduction in intensity. This quenching mechanism is widely employed by other nanosheet systems in a variety of biological and environmental sensing applications, particularly by quenching of dye-labelled single-stranded DNA for biomolecular detection.^60–63^

## Conclusions

We have demonstrated a simple process for the top-down exfoliation of layered hydrogen-bonded frameworks into ultrathin, high aspect ratio nanosheets with remarkable stability in water and sensing capabilities. Two frameworks incorporating amidinium and carboxylate synthons, either within a single mixed linker or as a co-crystal, were prepared using simple high-yielding syntheses. Single-crystal analysis show both systems formed layered materials held together in two-dimensions by strong, charge assisted hydrogen-bonding interactions, with only weak van der-Waal interlayer interactions. The systems underwent a “soft” liquid-phase ultrasonic exfoliation procedure to produce HONs with a thickness of 0.8 nm and micron sized lateral dimensions. The HONs exhibit remarkable stability in a range of highly competitive solvents, including maintaining structures after suspension in water at 80 °C for three days. This stability is particularly remarkable given that both components are highly water soluble and the nanosheets are only held together by strong hydrogen-bonds. The HONs also demonstrate the potential for use in sensing applications showing strong quenching of fluorescent dyes.

HOFs are a diverse class of materials with a variety of useful properties and promising applications. Exfoliation dramatically increases a materials surface area, exposes active sites, and aids incorporation within membranes and thin films unlocking distinct new opportunities for HONs. A wide range of extended and functionalised derivatives of **HON-1** and **HON-2** can readily be envisaged which could enable their use in a wide range of sensing, catalysis, separation, and electronics applications. We anticipate that this simple and scalable approach will be broadly applicable to a wide range of other layered HOFs, particularly those based on other strong hydrogen-bond donor–acceptor pairs.^45,64^ The remarkable stability of these hydrogen-bond based materials also has broad implications for the design of a wide range of other supramolecular materials.

## Author contributions

S. A. B. synthesized and characterized **1**. N. G. W. synthesized and characterized **2·TP**. J. N. performed exfoliation studies, nanosheet characterization, water stability tests, and quenching experiments. The project was conceived by N. G. W. and J. A. F. The manuscript was written by J. N. with editing by N. G. W. and J. A. F. All authors have given approval to the final version of the manuscript.

## Conflicts of interest

There are no conflicts to declare.

## Supplementary Material

SC-012-D0SC06906J-s001

SC-012-D0SC06906J-s002
