# Supplementary material for: Monolayer nanosheets formed by liquid exfoliation of charge-assisted hydrogen-bonded frameworks
Source: Chem Sci. 2021 Jan 14;12(9):3322–7. doi: 10.1039/d0sc06906j (PMC8179369; doi:10.1039/d0sc06906j)
Supplement: SC-012-D0SC06906J-s001 [file SC-012-D0SC06906J-s001.pdf]

Electronic Supplementary Material (ESI) for Chemical Science.
This journal is © The Royal Society of Chemistry 2021

Supporting Information

Monolayer nanosheets formed by liquid exfoliation of charge-assisted hydrogen bonded frameworks

Joshua Nicks,[a] Stephanie A. Boer,[b] Nicholas G. White*[b] and Jonathan A. Foster*[a]

a. Department of Chemistry, University of Sheffield, Sheffield, S3 7HF, UK

b. Research School of Chemistry, The Australian National University, Canberra, ACT 2600, Australia

Contents.

S1. Synthesis and Characterisation of **1** and **2·TP**

S2. Preparation of **HON-1** and **HON-2**

S3. Water Stability Experiments

S4. Quenching Studies

S5. References

S1. Synthesis and Characterisation of 1 and 2·TP

General remarks

Na_2·**TP** [S1] was prepared from benzene-1,4-dicarboxylic acid, and **2**·Cl_2 [S2] was prepared from 1,4-dicyanobenzene as previously described, dry THF was distilled from sodium. All other compounds were bought from commercial suppliers and used as received.

NMR spectra were collected on a Bruker Avance spectrometer and are referenced to the residual solvent signal.[S3] Infrared spectra were recorded on a Perkin-Elmer Spectrum Two FTIR spectrometer. Electrospray ionization (ESI) mass spectrometry data were acquired on a Micromass Waters ZMD spectrometer. Elemental analyses were recorded at London Metropolitan University. Thermogravimetric analyses were recorded on a TA Instruments Q500 analyzer. PXRD data for bulk material were recorded at room temperature using a PANalytical Empyrean diffractometer using Cu Kα radiation and a PIXcel detector.

1^{H}·Cl

A solution of 4-cyanobenzoic acid (0.735 g, 5.00 mmol) in dry THF (25 mL) was cooled in a dry ice/acetone bath under a nitrogen atmosphere. A solution of LiHMDS in THF (1.0 M, 15 mL, 15 mmol) was added dropwise, resulting in the immediate formation of a precipitate. The mixture was allowed to warm to room temperature and stirred overnight. The reaction was then cooled in an ice-bath and ethanolic HCl (prepared by cautiously adding 2 mL acetyl chloride to 10 mL ethanol in an ice-bath) was added dropwise, resulting in the formation of a pale precipitate. All volatiles were removed under reduced pressure and the crude product was suspended in water (20 mL), sonicated for 20 minutes and then the mixture filtered. The precipitate was again suspended in water (10 mL), sonicated for 10 minutes), filtered and then thoroughly dried to give **1^{H}**·Cl as a pale brown powder. Yield: 0.789 g (3.93 mmol, 79%).

^{1}H NMR (d_6-DMSO): 13.50 (br. s, 1H), 9.52 (s, 2H), 9.28 (s, 2H), 8.12 (d, *J* = 8.5 Hz, 2H), 7.92 (d, *J* = 8.5 Hz, 2H) ppm. ^{13}C NMR (d_6-DMSO): 166.3, 165.3, 135.2, 131.9, 129.5, 128.5 ppm. HRESI-MS (pos.): 165.0654, calc. for $[C_8H_9N_2O_2]^+$ (*i.e.* loss of Cl^-): 165.0659 Da. ATR-IR (*inter alia*): 1705, 1665 cm^{-1} (C=O, C=N stretches).

*The ^{1}H and ^{13}C NMR spectra of **1^{H}**·Cl are provided in Figures S1 and S2.*

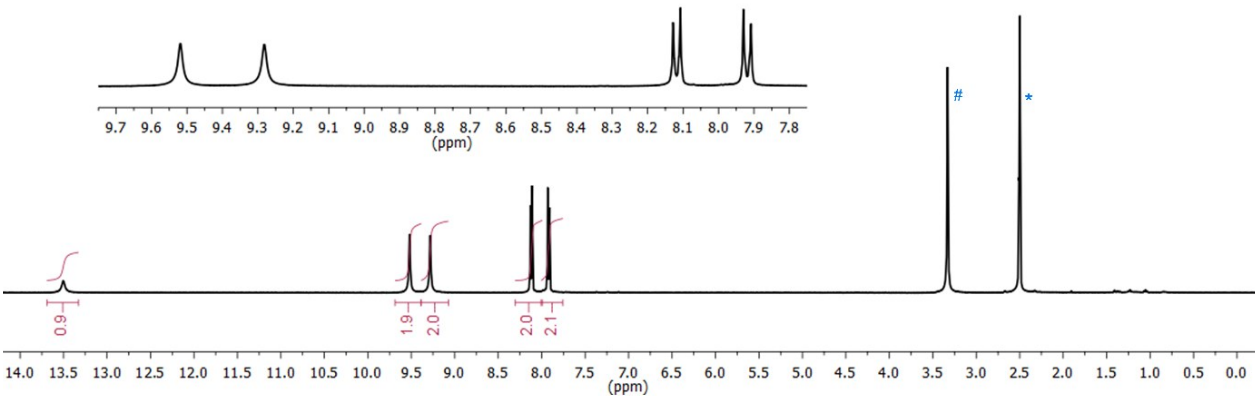

Figure S1. ^{1}H NMR spectrum of **1**$^{\mathbf{H}}$·Cl, residual solvent peak is marked * and water peak is marked # (d_6-DMSO, 298 K, 400 MHz).

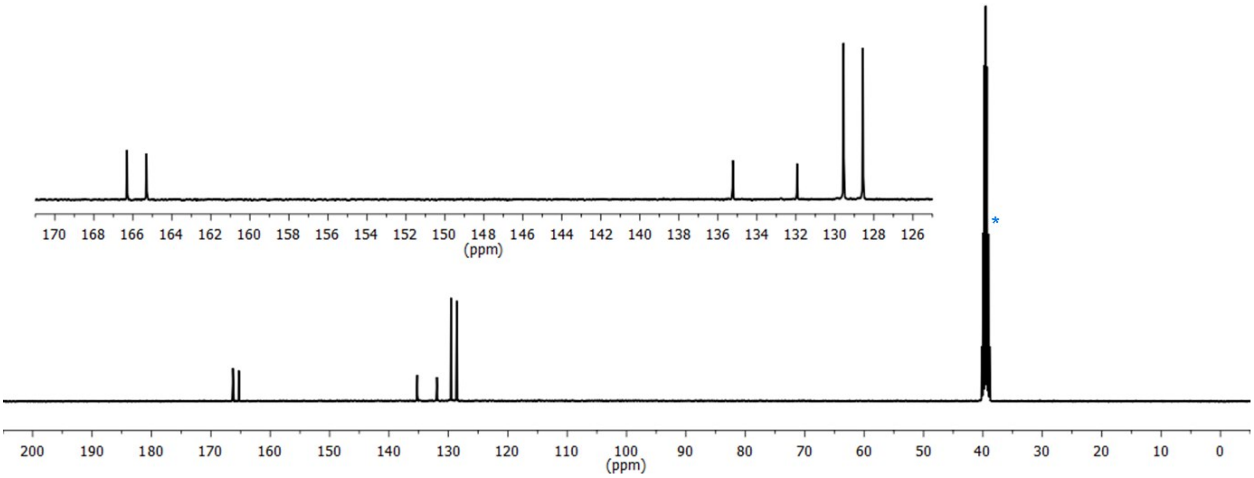

Figure S2. ^{13}C NMR spectrum of **1**$^{\mathbf{H}}$·Cl, residual solvent peak is marked * (d_6-DMSO, 298 K, 101 MHz).

1

The carboxylic acid-containing molecule **1^H·**Cl (0.250 g, 1.25 mmol) was dissolved in a DMF/water (1:1 v:v, 130 mL) and heated to 120 °C for 2 days, during which time colourless crystals formed. These were isolated by filtration, washed with water (5 × 10 mL) and dried *in vacuo* to give **1**. Yield: 0.155 g (0.944 mmol, 76%).

^{1}H NMR (d_6-DMSO containing 1 drop of 20% DCl in D_2O): 9.62* (s, 2H), 9.43* (s, 2H), 8.10 (d, *J* = 8.4 Hz, 2H), 7.94 (d, *J* = 8.1 Hz, 2H). ATR-IR (*inter alia*): 1703, 1583 cm^{-1} (C=O, C=N stretches). EA: C 58.4, H 4.8, N. 17.0%, calc. for [$C_8H_8N_2O_2$]: C 58.5, H 4.9, N 17.1%.

*These peaks integrate to a value lower than expected due to H/D exchange.

*The ^{1}H NMR spectrum of acid-digested **1** is provided in Figure S3.*

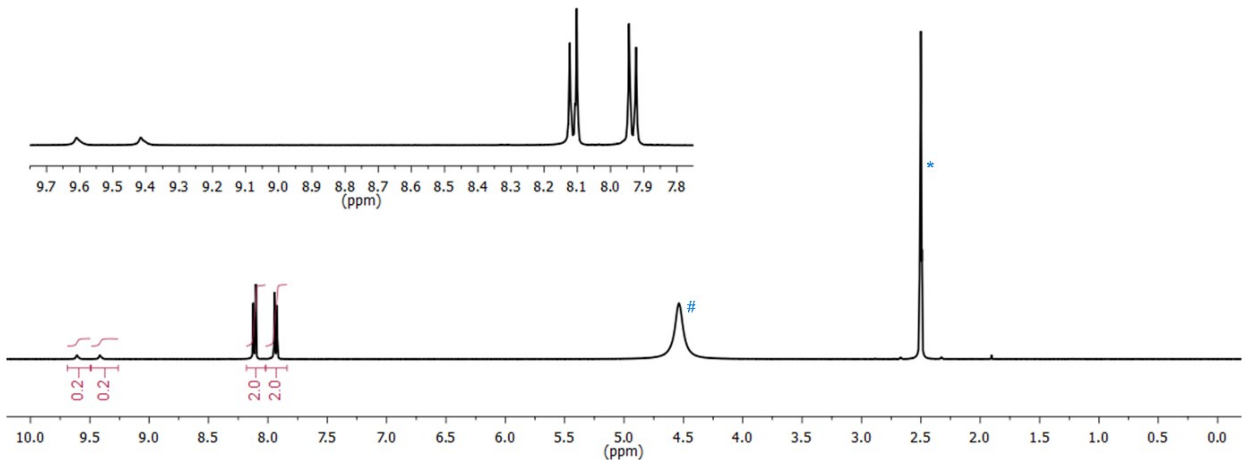

Figure S3. ^{1}H NMR spectrum of acid-digested **1**, residual solvent peak is marked * and water peak is marked # (d_6-DMSO containing 20% DCl in D_2O, 298 K, 400 MHz). Note that the amidinium N–H resonances integrate to a much lower value than expected (2H each) due to H/D exchange.

The PXRD trace of bulk **1** is shown in Figure S4. Good agreement is observed between the measured data and that calculated from the room temperature SCXRD structure.

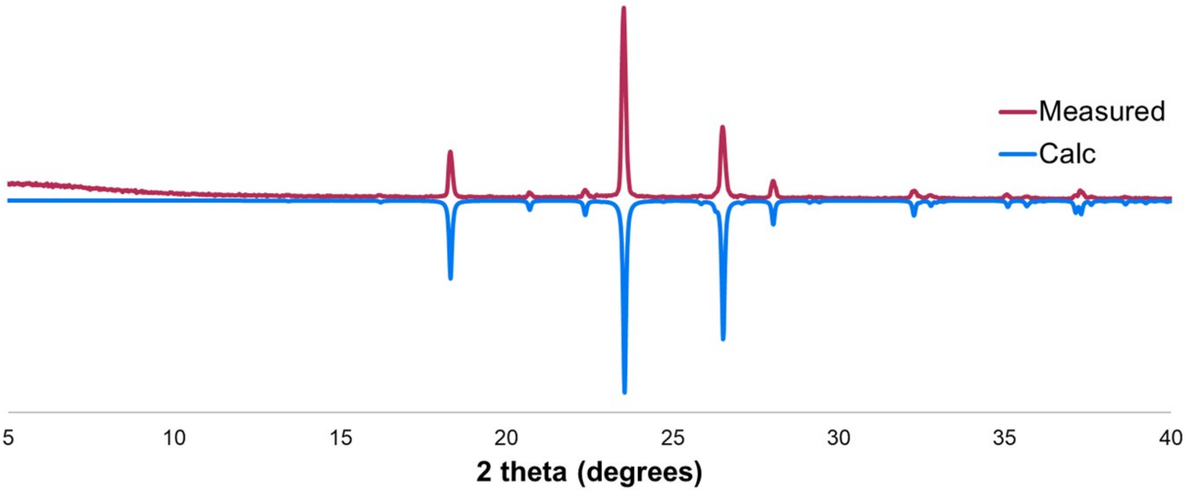

Figure S4. Measured PXRD trace of **1** (up, maroon) and comparison with that calculated from SCXRD data (down, blue).

The TGA trace of bulk **1** is shown in Figure S5. No mass loss is seen until > 300 °C, at which point thermal decomposition is observed.

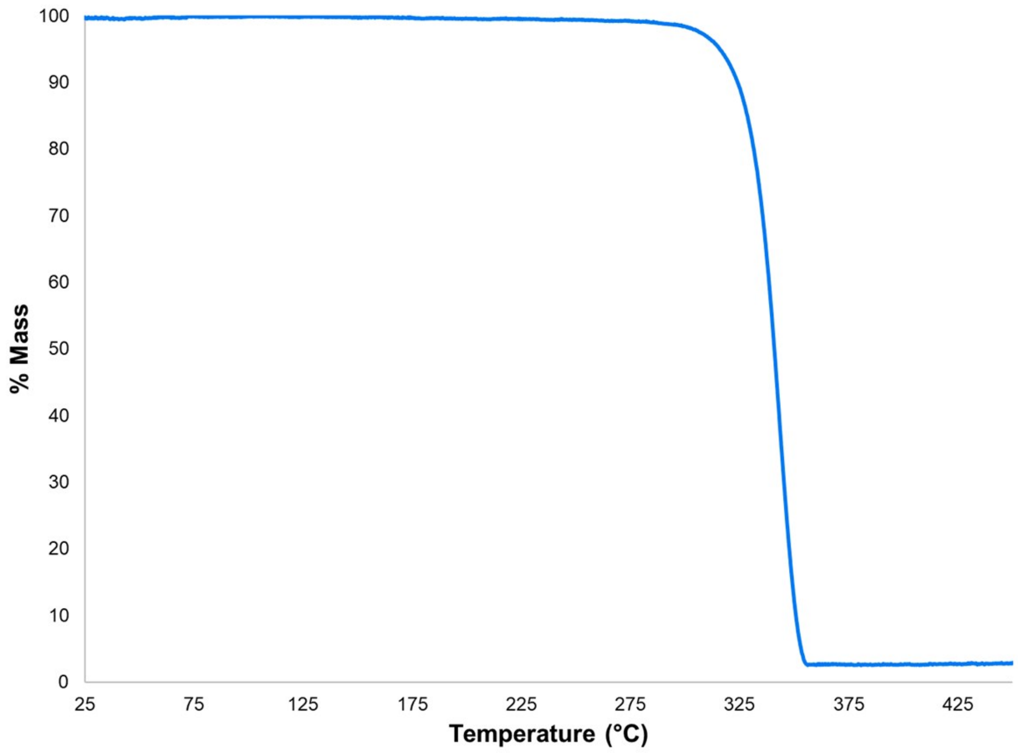

Figure S5. TGA trace of **1** (recorded under N_2 at a ramp rate of 5 °C/minute).

2·TP

*We have previously reported the synthesis of this framework on a small (20 mg scale) from **2**·Cl_2 and tetrabutylammonium terephthalate.*[S4] *The procedure was optimized to allow preparation of a larger sample of material. In this procedure, we have used sodium terephthalate as it is cheaper and easier to prepare than tetrabutylammonium terephthalate.*

A solution of bis(amidinium) **2·Cl_2** (0.235 g, 1.00 mmol) in water (50 mL) was placed in a measuring cylinder. It was carefully layered with water (50 mL), and then sodium terephthalate (0.210 g, 1.00 mmol) in water (50 mL). After several days, the resulting white microcrystalline solid was isolated by filtration, washed with water (4 × 10 mL) and methanol (4 × 10 mL), and dried thoroughly to give **2·TP**. Yield: 0.293 g (0.892 mmol, 89%).

^{1}H NMR (Figure S6) and PXRD data (Figure S7) were consistent with those previously reported.[S4] *Good agreement is observed between the measured PXRD data and that calculated from the room temperature SCXRD structure.*

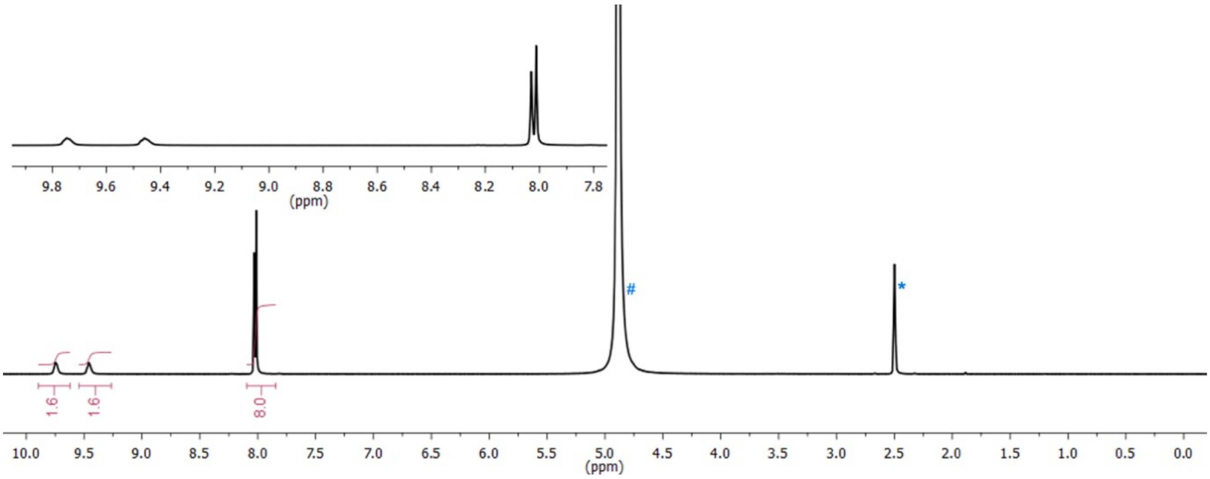

Figure S6. ^{1}H NMR spectrum of acid-digested **2·TP**, residual solvent peak is marked * and water peak is marked # (d_6-DMSO containing 20% DCl in D_2O, 298 K, 400 MHz). Note that the amidinium N–H resonances integrate to a much lower value than expected (2H each) due to H/D exchange.

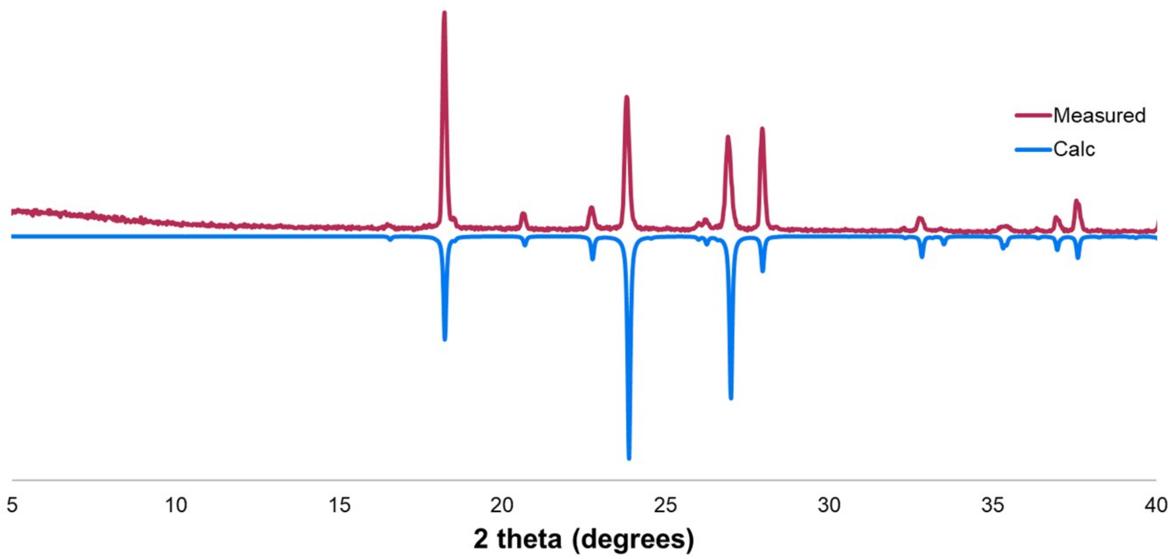

Figure S7. Measured PXRD trace of **2·TP** (up, maroon) and comparison with that calculated from SCXRD data (down, blue).

SCXRD studies

Details of data collection and refinement

We have obtained two single crystal structures of **1**. The first was collected at the Australian Synchrotron at 100 K, and due to the weakly-diffracting crystal and limitations of the beamline has poor data completeness. We subsequently re-collected this structure on a home-source diffractometer at room temperature, using a better quality crystal. The data for the structure collected at room temperature are superior to those collected at 100 K. All figures, details of bond lengths and comparisons with PXRD data use the 293 K collection, however we have included both structures for completeness. We have also collected a structure of **2·TP** at 293 K, having previously reported a low temperature (150 K) structure of this compound.[S4]

Data were either collected using mirror-monochromated Cu Kα radiation on an Agilent SuperNova diffractometer at 293 K or using beamline MX1[S5] of the Australian Synchrotron at 100 K. Raw frame data for the structures collected at 293 K (including data reduction, interframe scaling, unit cell refinement and absorption corrections) were processed using CrysAlis Pro,[S6] while data for the structure collected at 100 K were processed using XDS.[S7]

All structures were solved using Superflip,[S8] and refined using full-matrix least-squares on F^2 within the Crystals suite.[S9] All non-hydrogen atoms were refined with anisotropic displacement parameters. All hydrogen atoms were clearly visible in the Fourier difference map: C–H hydrogen atoms were initially refined with restraints on bond lengths and angles, after which the positions were used as the basis for a riding model.[S10] The positions of N–H hydrogen atoms were refined with restraints on N–H and C–N–H angles.

Apart from restraints on the hydrogen atom positions, it was not necessary to use any crystallographic restraints in any of the refinements.

Crystallographic data for the structures reported in this paper (**1** at 100 K and 293 K, **2·TP** at 293 K) have been deposited with the Cambridge Crystallographic Data Centre (CCDC Numbers: 2047425–2047427). Thermal ellipsoid plots are shown in Figures S8–S10, and selected data are summarized in Table S1. Data for the previously-published[S4] low temperature structure of **2·TP** are also available from the CCDC (CSD reference code: ZEFSOV, CSD deposition number: 1537445).

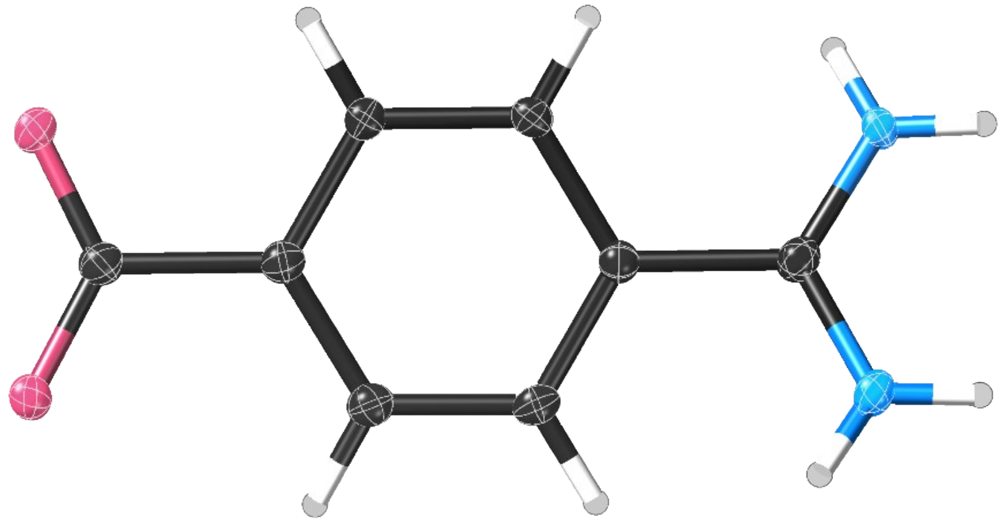

Figure S8. Thermal ellipsoid plot of the asymmetric unit of **1** at 100 K, ellipsoids shown at 50% probability level.

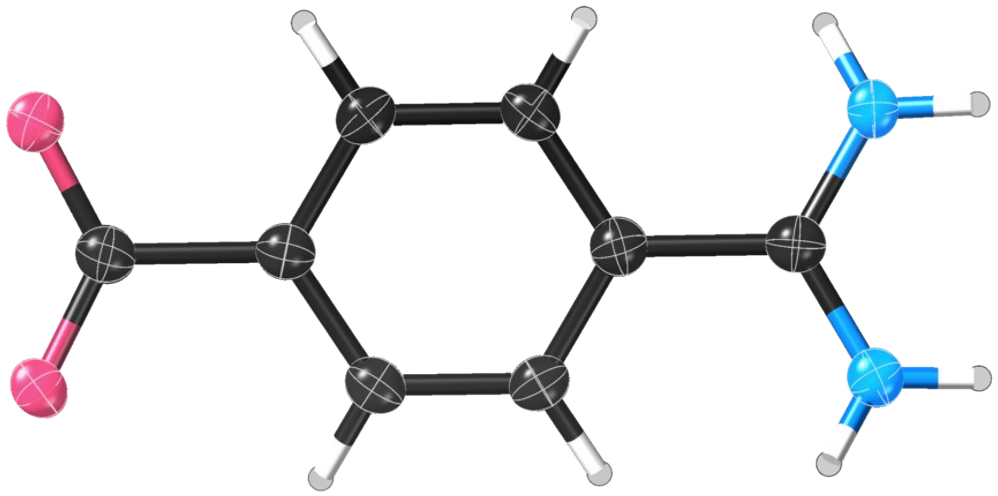

Figure S9. Thermal ellipsoid plot of the asymmetric unit of **1** at 293 K, ellipsoids shown at 50% probability level.

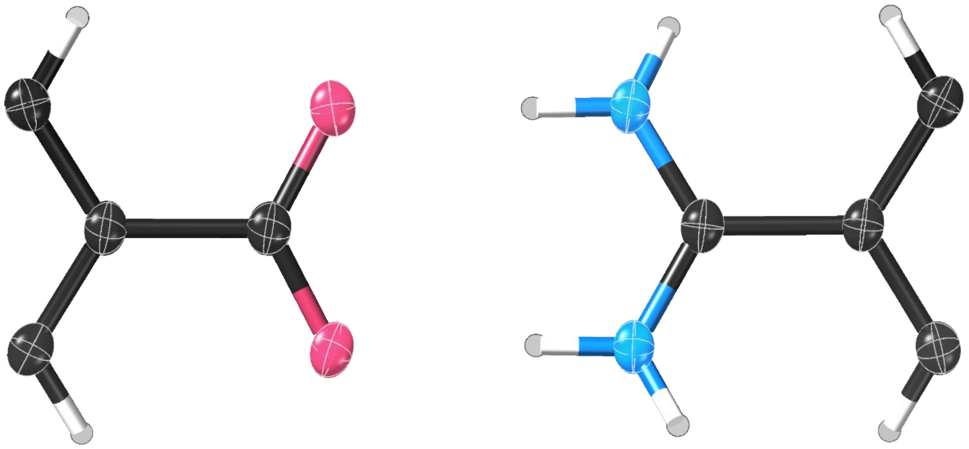

Figure S10. Thermal ellipsoid plot of the asymmetric unit of **2·TP** at 293 K, ellipsoids shown at 50% probability level.

Table S1. Selected crystallographic data.

	1 (100 K)	1 (293 K)	2_TP (293 K)
Radiation type	Synchrotron (λ = 0.7108 Å)	Cu Kα (λ = 1.54184 Å)	Cu Kα (λ = 1.54184 Å)
Temperature (K)	100	293	293
Formula	$C_8H_8N_2O_2$	$C_8H_8N_2O_2$	$(C_8H_{12}N_4)_{0.5}\cdot(C_8H_4O_4)_{0.5}$
Formula weight	164.16	164.16	164.16
a (Å)	6.9310(14)	7.03480(2)	5.5557(5)
b (Å)	7.5900(15)	7.85120(2)	7.8957(7)
c (Å)	7.7350(15)	7.88270(2)	9.0549(8)
α (°)	60.94(3)	61.268(5)	71.386(8)
β (°)	82.44(3)	74.309(4)	83.555(7)
γ (°)	75.12(3)	82.320(5)	74.217(7)
Unit cell volume ($Å^3$)	343.73(16)	367.540(19)	362.09(6)
Crystal system	triclinic	triclinic	triclinic
Space group	P–1	P–1	P–1
Z	2	2	2
Reflections (all)	3285	6926	5714
Reflections (unique)	1666	1434	1452
Min/max difference density (e A^{-3})	–0.79/0.68	–0.37/0.36	–0.20/0.25
R_{int}	0.037	0.029	0.024
R_1 [$I > 2\sigma(I)$]	0.093	0.072	0.046
$wR_2(F^2)$ (all data)	0.331	0.252	0.138
CCDC number	2047425	2047426	2047427

Effect of temperatures on structures

Increasing the temperature (from 100 K to 293 K for **1**, and from 150 K to 293 K for **2·TP**) causes small increases in the unit cell axes (0.2–3.4%) as well as small changes in the unit cell angles (up to ±1.1%). This results in an increase in unit cell volume of 6.9% for **1** and 1.7% for **2·TP**. Given the relatively low resolution of hydrogen atom positions in X-ray crystal structures, we have not speculated on the effect of temperature on hydrogen bonding parameters.

SEM Imaging of 1 and 2·TP

SEM images of **1** and **2·TP** are shown in Figures S12 and S13 at multiple magnifications.

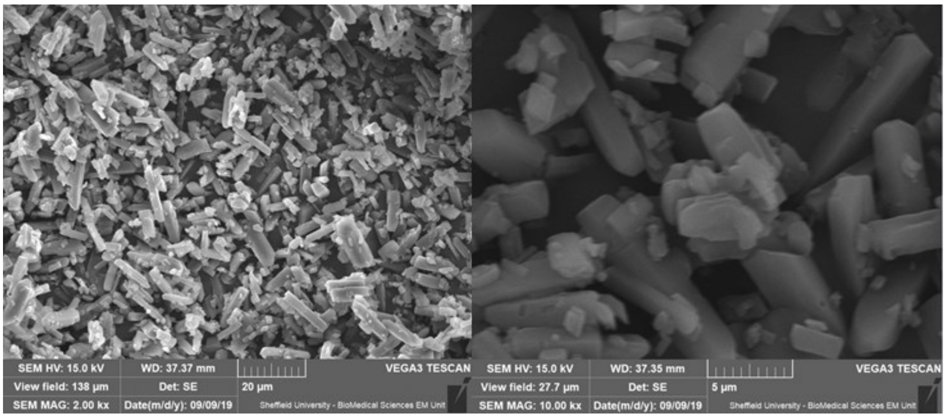

Figure S11. SEM images at 20 μm and 5 μm scales, indicating the layered morphology of **1**.

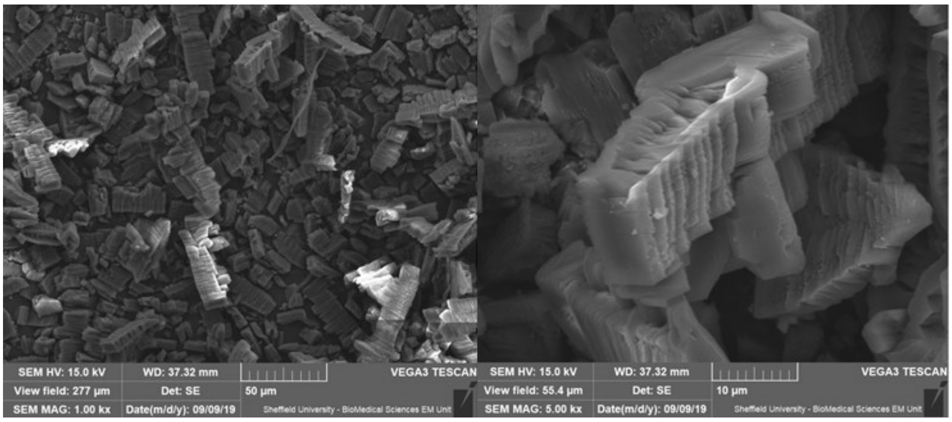

Figure S12. SEM images at 50 μm and 10 μm scales, indicating the layered morphology of **2·TP**.

S2. Preparation of HON1 and HON2

General Remarks

Atomic force microscopy images were recorded using a Bruker Multimode 5 atomic force microscope, operating in soft-tapping mode in air under standard ambient temperature and pressure, fitted with Bruker OTESPA-R3 silicon cantilevers operated with a drive amplitude of ~18.70 mV and resonance frequency of ~236 kHz. Samples were prepared by drop-casting 10 µL drops of suspension onto the centre of freshly cleaved mica sheets heated to 100 °C on a hot plate. These sheets were stuck to stainless steel, magnetic Agar scanning probe microscopy specimen discs. Images were processed using Gwyddion software.

DLS data were collected using a Malvern Zetasizer Nano Series particle size analyser, using a He-Ne laser at 633 nm, operating in backscatter mode (173 °). Samples were placed in quartz cuvettes and equilibrated at 298 K for 60 s prior to analysis.

Powder X-ray diffraction patterns were collected using a Bruker-AXS D8 diffractometer, using Cu K_α (λ=1.5418 Å) radiation, operating at 40 V and 40 mA, and fitted with an energy-dispersive LynxEye position sensitive detector in Bragg Brentano parafocussing geometry using a flat silicon plate.

Ultrasonic Exfoliation

Samples of **1** or **2·TP** were suspended in DMF or acetone respectively (5 mL) and vortexed for 20 s, followed by sonication for 12 hr using a Fisher brand Elmasonic P 30H ultrasonic bath operating at 80 kHz and 100% power (320 W). Samples were rotated using an overhead stirrer to ensure even exposure and prevent the occurrence of “hot spots”, and the bath was fitted with a water coil to maintain temperature at approximately 18–21 °C. After sonication, samples were transferred into polypropylene centrifuge tubes and centrifuged for 1 hr at 1500 rpm, in order to sediment unexfoliated material and leave **HON-1** and **HON-2** in suspension.[S11]

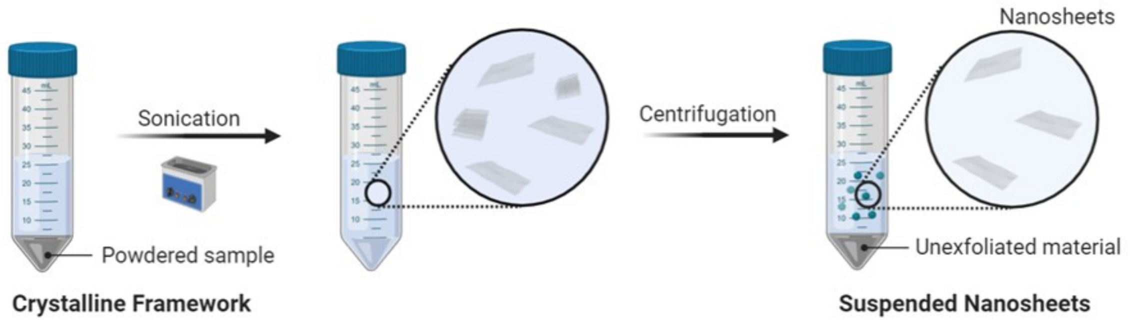

Figure S13. Illustration of the exfoliation of layered hydrogen bonded frameworks into nanosheets usind liquid-assisted sonication.

Nanosheet Characterisation

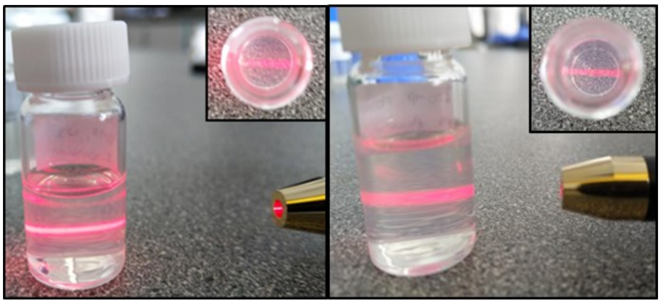

Figure S14. Tyndall scattering effects exhibited by as prepared suspensions HON1 in DMF (left) and HON2 in acetone (right).

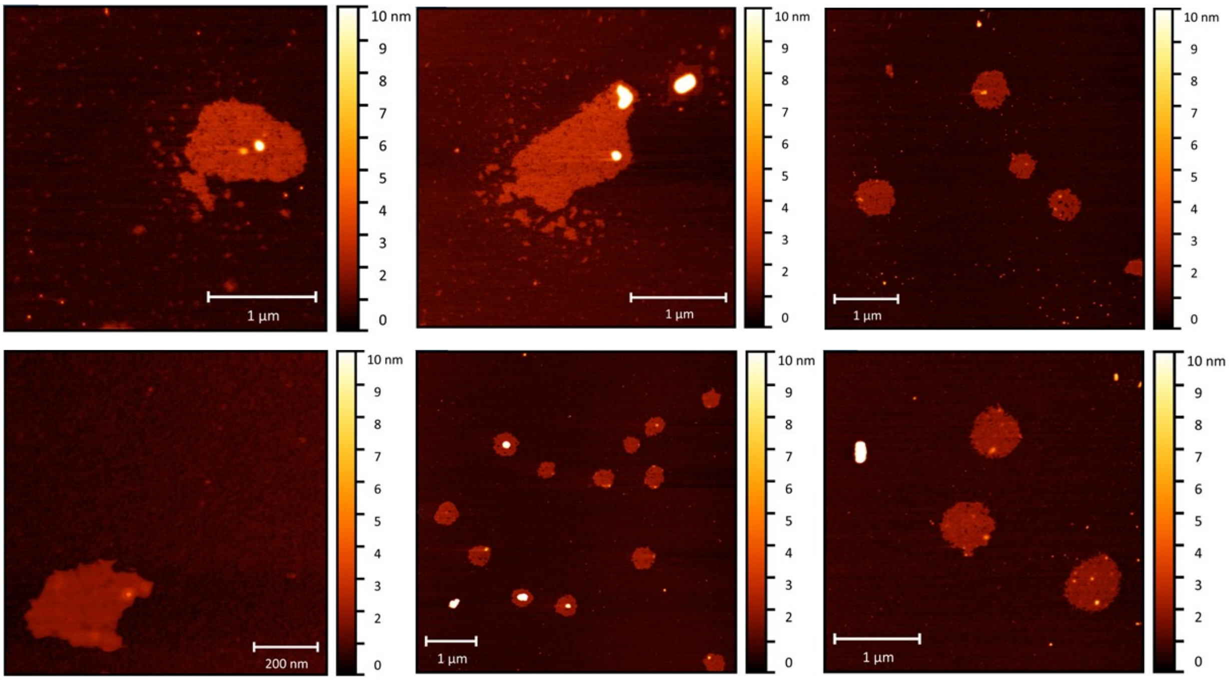

Figure S15. AFM topographical images of **HON1** nanosheets.

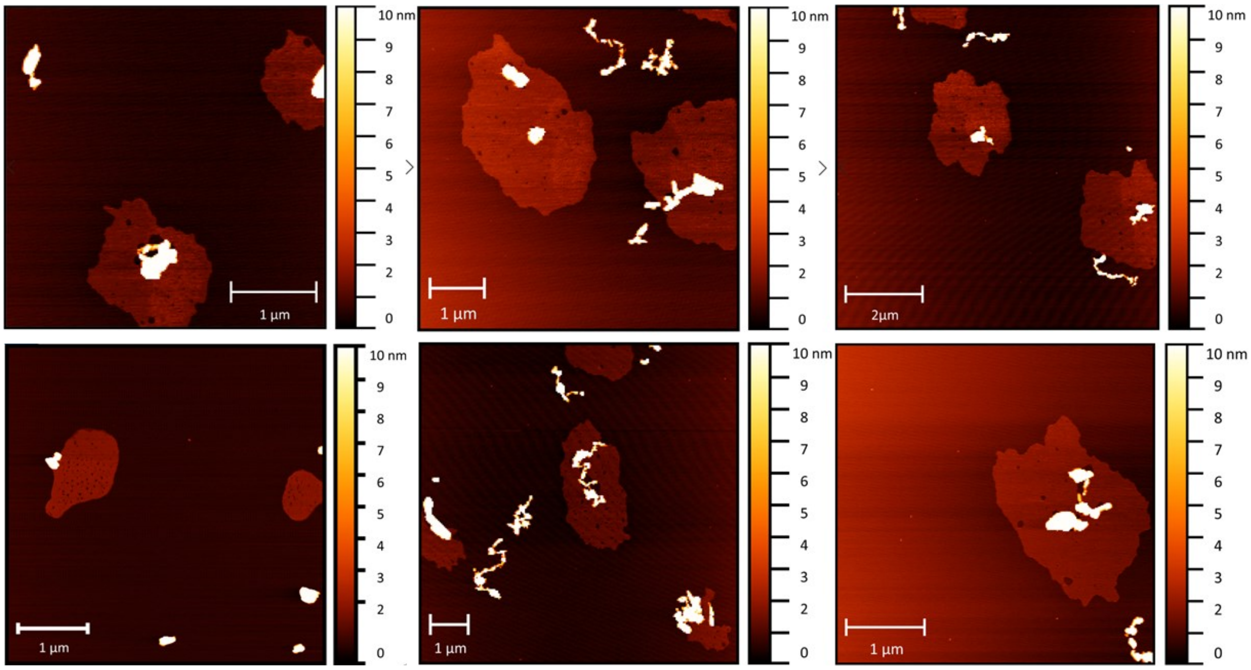

Figure S16. AFM topographical images of **HON2** nanosheets.

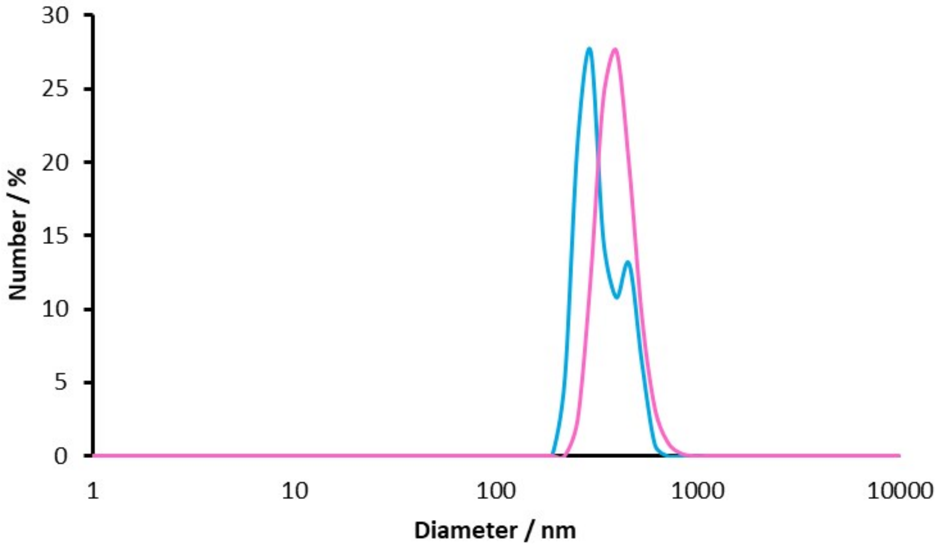

Figure S17. DLS number plots for suspensions of **HON-1** (blue) and **HON-2** (pink).

S3. Water Stability Experiments

Samples of both HON-1 and HON-2 were treated with distilled water (10 mL) and heated for 3 days at 80 °C in sealed reaction vials. HONs were then reisolated by centrifugation at 10000 rpm for 1 hour, washed with acetone to remove residual water, then subject to XRD and AFM analysis.

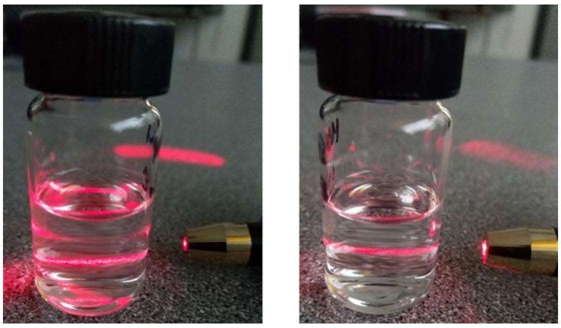

Figure S18. Tyndall scattering effects exhibited by suspensions of **HON-1** (left) and **HON-2** (right) after treatment in water.

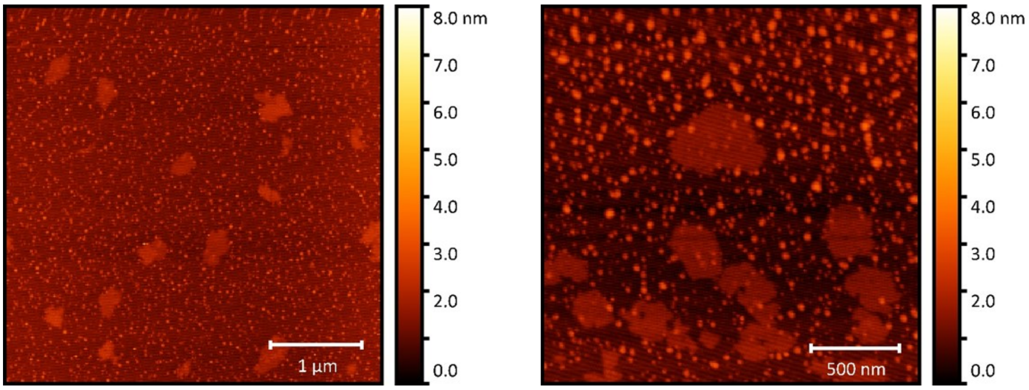

Figure S19. AFM topographical images of **HON-1** nanosheets after water treatment.

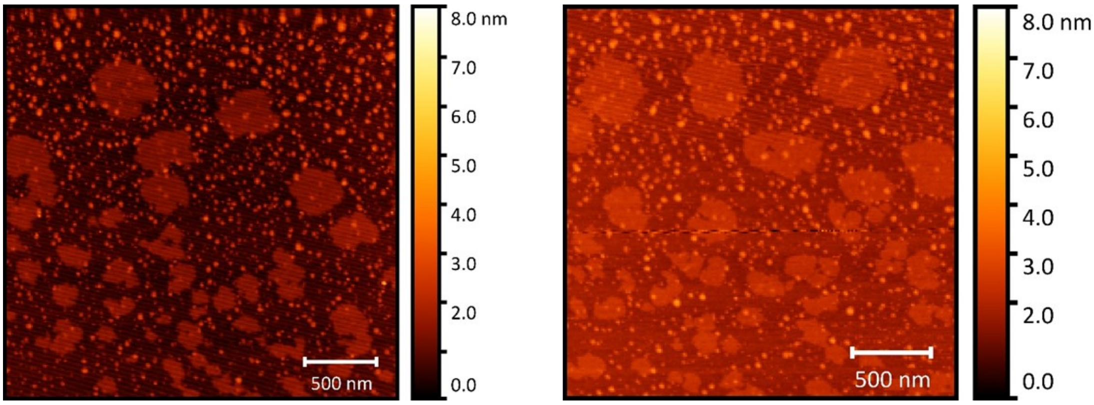

Figure S20. AFM topographical images of **HON-2** nanosheets after water treatment.

S4. Quenching Studies

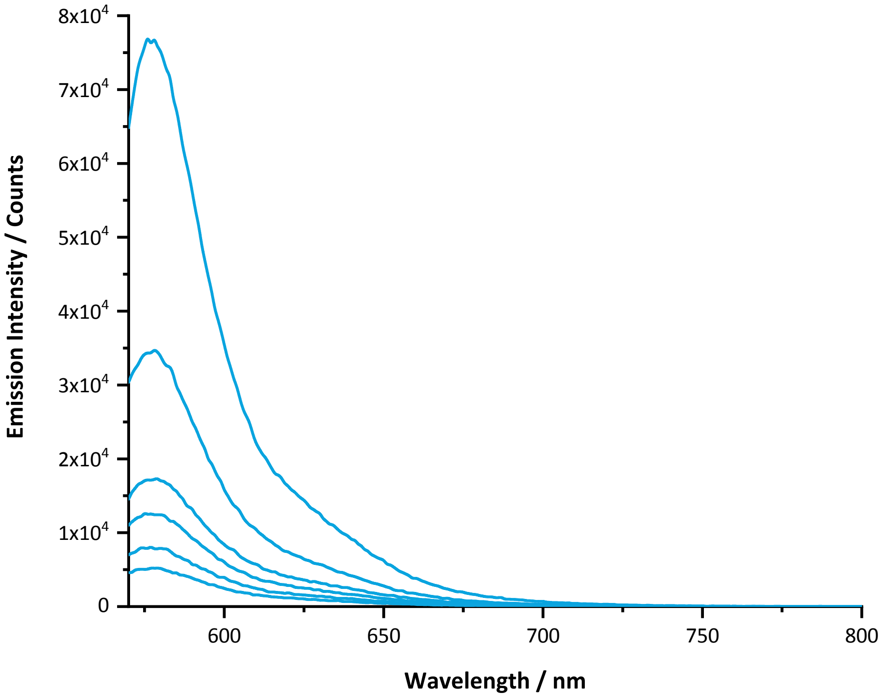

Figure S21. Emission spectra of Rhodamine B (top) in acetone with increasing quantities of HON-1 nanosheets (0.01 mg each).

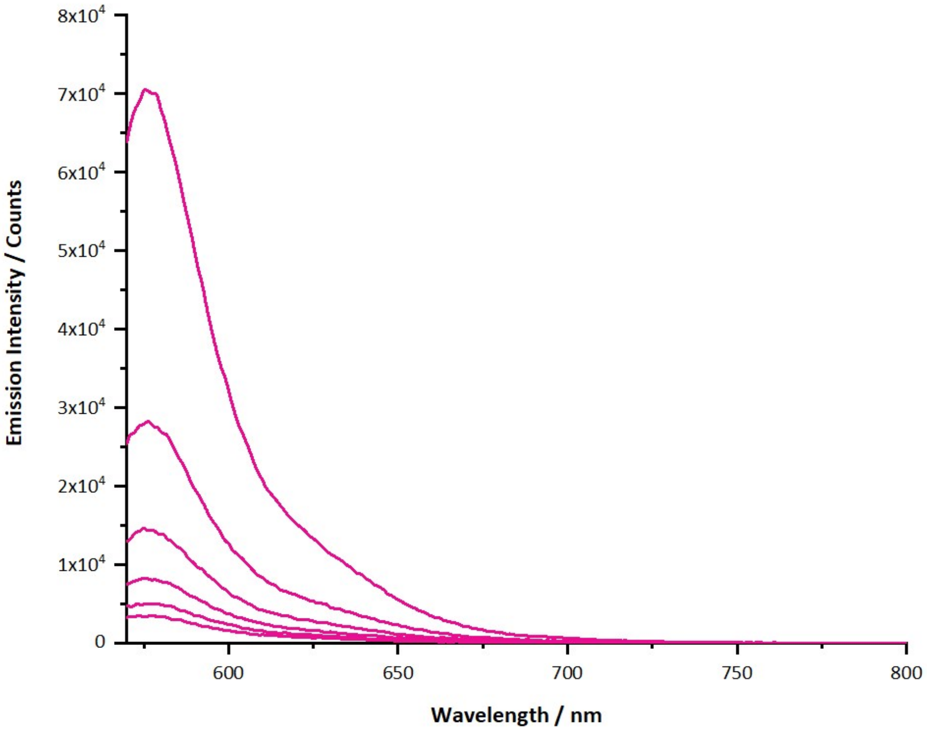

Figure S22. Emission spectra of Rhodamine B (top) in acetone with increasing quantities of HON-2 nanosheets (0.01 mg each).

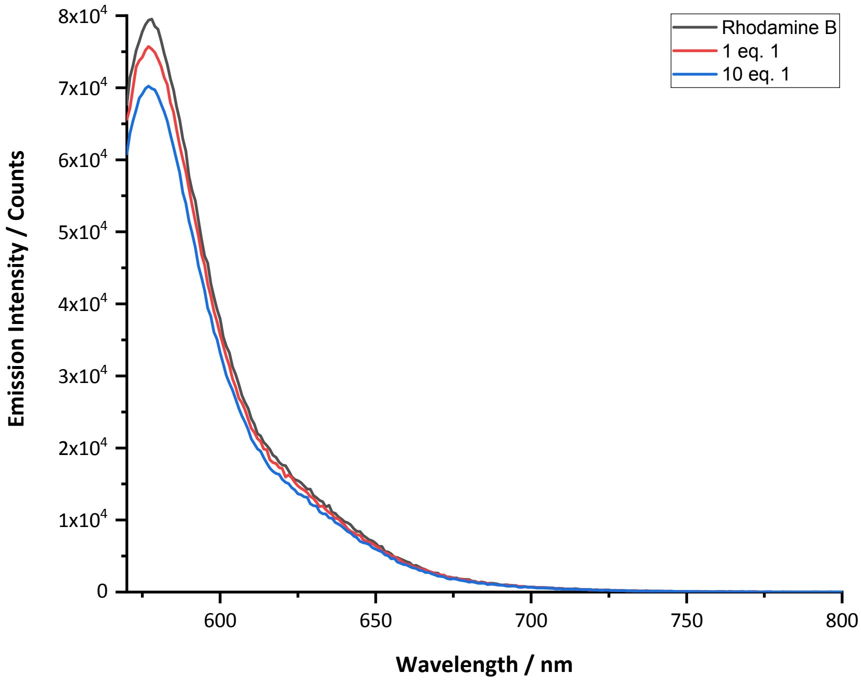

Figure S23. Emission spectra of Rhodamine B in acetone with increasing quantities of bulk framework **1**.

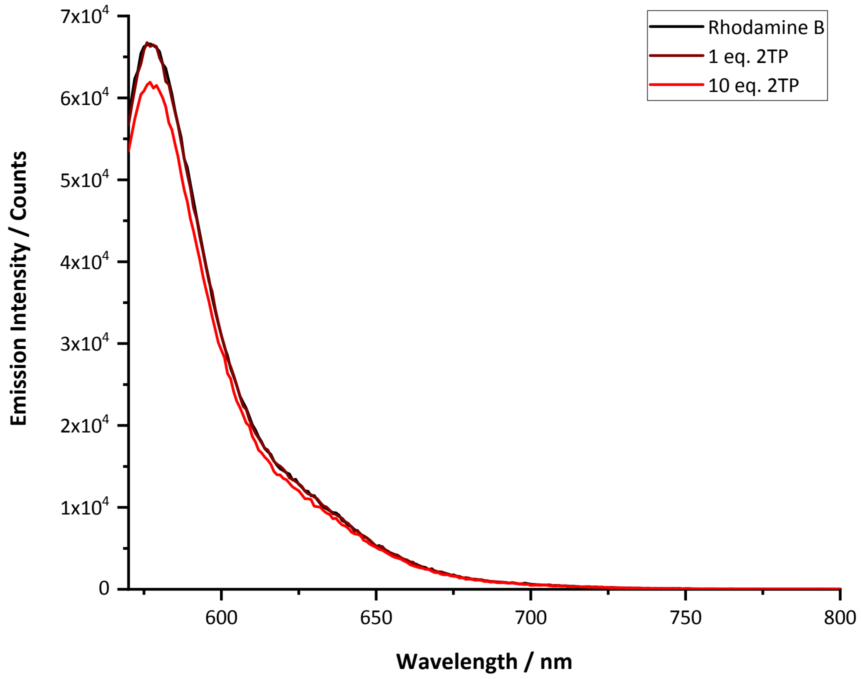

Figure S24. Emission spectra of Rhodamine B in acetone with increasing quantities of bulk framework **2TP**.

S5. References

[S1] M-F. Zaltariov, A. Vlad, M. Cazacu, S. Shova, M. Balan, C. Racles, A novel siloxane-containing dicarboxylic acid, 1,3-bis(*p*-carboxyphenylene-ester-methylene)tetramethyldisiloxane, and its derivatives: ester macrocycle and supramolecular structure with a copper complex, *Tetrahedron*, **2014**, *70*, 2661–2668.

[S2] K. Wang, L-M. Yang, X. Wang, L. Guo, G. Cheng, C. Zhang, S. Jin, B. Tan, A. Cooper, Covalent triazine frameworks via a low-temperature polycondensation approach, *Angew. Chem. Int. Ed.* **2017**, *56*, 14149–14513.

[S3] H. E. Gottlieb, V. Kotlyar, A. Nudelman, NMR chemical shifts of common laboratory solvents as trace impurities, *J. Org. Chem.* **1997**, *62*, 7512–7515.

[S4] M. Thomas, T. Anglim Lagones, M. Judd, M. Morshedi, M. L. O'Mara, N. G. White, Hydrogen bond-driven self–assembly between amidiniums and carboxylates: a combined MD, NMR and SCXRD study, *Chem. Asian J.* **2017**, *12*, 1587–1597.

[S5] N. P. Cowieson, D. Aragao, M. Clift, D. J. Ericsson, C. Gee, S. J. Harrop, N. Mudie, S. Panjikar, J. R. Price, A. Riboldi-Tunnicliffe, R. Williamson, T. Caradoc-Davies, MX1: a bending-magnet crystallography beamline serving both chemical and macromolecular crystallography communities at the Australian Synchrotron, *J. Synchrotron Radiat.* **2015**, *1*, 187–190.

[S6] Agilent Technologies, *CrysAlisPro*, **2011**.

[S7] W. Kabsch, Automatic processing of rotation diffraction data from crystals of initially unknown symmetry and cell constants, *J. Appl. Cryst.* **1993**, *26*, 795–800.

[S8] L. Palatinus, G. Chapuis, *SUPERFLIP* – a computer program for the solution of crystal structures by charge flipping in arbitrary dimensions, *J. Appl. Cryst.* **2007**, *40*, 786–790.

[S9] P. W. Betteridge, J. R. Carruthers, R. I. Cooper, K. Prout, D. J. Watkin, *CRYSTALS* version 12: software for guided crystal structure analysis, *J. Appl. Cryst.* **2003**, *36*, 1487.

[S10] R. I. Cooper, A. L. Thompson, D. J. Watkin, *CRYSTALS* enhancements: dealing with hydrogen atoms in refinement, *J. Appl. Cryst.* **2010**, *43*, 1100–1107.

[S11] D. J. Ashworth, A. Cooper, M. Trueman, R. W. M. Al-Saedi, S. D. Liam, A. J. Meijer and J. A. Foster, Chem. - A Eur. J., **2018**, *24*, 17986-17996.
